# Angular Velocities and Linear Accelerations Derived from Inertial Measurement Units Can Be Used as Proxy Measures of Knee Variables Associated with ACL Injury

**DOI:** 10.3390/s22239286

**Published:** 2022-11-29

**Authors:** Holly S. R. Jones, Victoria H. Stiles, Jasper Verheul, Isabel S. Moore

**Affiliations:** 1 Cardiff School of Sport and Health Sciences, Cardiff Metropolitan University, Cyncoed Campus, Cardiff CF23 6XD, UK; 2 Sport and Health Sciences, University of Exeter, St Luke’s Campus, Exeter EX1 2LU, UK

**Keywords:** wearable technologies, injury monitoring, knee biomechanics, injury risk, field-based assessments, gyroscope, accelerometer

## Abstract

Given the high rates of both primary and secondary anterior cruciate ligament (ACL) injuries in multidirectional field sports, there is a need to develop easily accessible methods for practitioners to monitor ACL injury risk. Field-based methods to assess knee variables associated with ACL injury are of particular interest to practitioners for monitoring injury risk in applied sports settings. Knee variables or proxy measures derived from wearable inertial measurement units (IMUs) may thus provide a powerful tool for efficient injury risk management. Therefore, the aim of this study was to identify whether there were correlations between laboratory-derived knee variables (knee range of motion (RoM), change in knee moment, and knee stiffness) and metrics derived from IMUs (angular velocities and accelerations) placed on the tibia and thigh, across a range of movements performed in practitioner assessments used to monitor ACL injury risk. Ground reaction forces, three-dimensional kinematics, and triaxial IMU data were recorded from nineteen healthy male participants performing bilateral and unilateral drop jumps, and a 90° cutting task. Spearman’s correlations were used to examine the correlations between knee variables and IMU-derived metrics. A significant strong positive correlation was observed between knee RoM and the area under the tibia angular velocity curve in all movements. Significant strong correlations were also observed in the unilateral drop jump between knee RoM, change in knee moment, and knee stiffness, and the area under the tibia acceleration curve (r_s_ = 0.776, r_s_ = −0.712, and r_s_ = −0.765, respectively). A significant moderate correlation was observed between both knee RoM and knee stiffness, and the area under the thigh angular velocity curve (r_s_ = 0.682 and r_s_ = −0.641, respectively). The findings from this study suggest that it may be feasible to use IMU-derived angular velocities and acceleration measurements as proxy measures of knee variables in movements included in practitioner assessments used to monitor ACL injury risk.

## 1. Introduction

Non-contact anterior cruciate ligament (ACL) ruptures are one of the most common and severe injuries in multidirectional field sports [1,2]. Athletes who sustain an ACL rupture typically undergo ACL reconstruction surgery in the hope of returning to their pre-injury level of sport [3,4]. However, return to sport following ACL reconstruction significantly increases the risk of sustaining a secondary ACL injury [5,6,7]. Specifically, when returning to sports that include frequent cutting and pivoting following ACL reconstruction, an athlete has a 3.9-fold increased risk of sustaining an ipsilateral ACL injury and a 5-fold increased risk of sustaining a contralateral ACL injury [8]. Monitoring ACL injury risk is crucial for practitioners to identify and target potential deficits associated with ACL injury. However, a major limitation for practitioners when monitoring ACL injury risk is the inability to assess an athlete in applied field-based settings.

Monitoring an athlete’s ACL injury risk in the field is challenging, primarily due to the limited ability to accurately measure an athlete’s movement and loading strategies in their natural training and/or competition environment [9]. In an attempt to overcome this, wearable inertial measurement unit (IMU) measurements, such as angular velocities and linear accelerations, have been used to quantify human motion during gait [10,11,12], single-leg landing tasks [13], and bilateral and unilateral drop jumps [14]. During the braking phase of landing in particular, changes in knee range of motion (RoM), external knee flexion moment, and knee stiffness have all been proposed to influence ACL injury risk [15,16,17]. Although gold-standard laboratory-based systems (i.e., marker-based three-dimensional motion capture and force plates) can be used to accurately measure these variables, these systems are time-consuming, and require costly equipment and skilled personnel, thus, they are not accessible to most practitioners. From a practitioner’s perspective, the use of wearable IMUs may provide a more accessible alternative to the current gold-standard analysis, i.e., marker-based three-dimensional motion capture and force plates, for monitoring ACL injury risk in the field.

Previous studies have examined the relationship between knee moments and angular velocities obtained from IMUs placed on the tibia and thigh. A significant strong positive correlation was found between peak knee extensor moment and tibial angular velocity during walking following ACL reconstruction [18]. Pratt and Sigward [13] also observed a significant moderate positive correlation between peak resultant knee moment and peak thigh angular velocity during a single-leg landing task in individuals following ACL reconstruction. As knee moment is a component of knee stiffness, a higher knee moment would produce greater knee stiffness for a given RoM, thus, correlations may also exist between knee stiffness and both the tibia and thigh angular velocities. Compared with non-injured controls, reduced knee stiffness was observed during a bilateral drop jump and 90° cutting manoeuvre in patients who had undergone ACL reconstruction surgery [17], which may be associated with an increased risk of sustaining a secondary ACL injury. Additionally, Milner et al. [19] found a strong negative correlation between knee RoM (another component of knee stiffness) and peak tibial axial accelerations, as well as a positive but weak correlation between knee stiffness and peak tibial axial accelerations during the initial loading phase of running in healthy participants. Further research is therefore needed to determine if relationships exist between knee variables associated with ACL injury and IMU angular velocity and acceleration metrics during movements performed in practitioner assessments used to monitor ACL injury risk.

To replicate the movements preceding a non-contact ACL injury, such as landing from a jump or change of direction manoeuvres combined with deceleration [1], assessments used by practitioners to monitor ACL injury risk often require an athlete to perform bilateral drop jumps, unilateral drop jumps, and cutting manoeuvres [20]. To provide representative (stable) data for each movement whilst reducing the burden of testing on performers, athletes typically perform three trials of each movement, with the mean result taken for each biomechanical variable [21,22,23]. Developing IMU-derived proxy measures of knee variables that can be incorporated into existing and accepted protocols used by practitioners to assess ACL injury risk, and that place no further burden on the participant, is key for enhancing the future use and acceptability of the method in the field. Research is therefore needed to confirm whether IMU-derived proxy measures for knee variables based on the mean of three movement trials (existing ACL risk-monitoring protocols) provides sufficiently stable data across all selected movements, compared to a higher number of trials. As efforts have recently been made to identify opportunities to reduce the burden of testing and improve the efficiency of practitioner assessments used to monitor ACL injury risk [24], determining whether IMU-derived proxy measures of knee variables associated with ACL injury can be acquired with a relatively small number of IMUs should remain a priority to aid practitioner convenience and expense.

Given the high rates of both primary and secondary ACL injuries in multidirectional field sports, there is a need to develop easily accessible IMU-derived metrics that can be used as proxy measures to assess knee variables associated with ACL injury. This may facilitate the ability of practitioners to monitor ACL injury risk more precisely in the field. Therefore, the aim of this study was to identify the strength of the correlations between laboratory-derived knee variables associated with ACL injury risk (knee RoM, change in knee moment, and knee stiffness) and metrics derived from IMUs (angular velocities and accelerations) placed on the tibia and thigh during movements performed in standard assessments to monitor ACL injury risk (bilateral and unilateral drop jumps, and a cutting manoeuvre). To confirm whether mean IMU-derived metrics calculated from three trials (existing ACL risk-monitoring protocols) provided stable data, correlations between the knee variables and mean IMU-derived metrics from three and five trials were compared for all movements. Finally, if a relationship was found between the knee variables and IMU-derived metrics, this study identified the location of the IMU (tibia or thigh) which demonstrated the strongest correlations.

## 2. Materials and Methods

### 2.1. Participants

Nineteen male multidirectional field sport athletes (i.e., football, rugby union, and American football) aged between 18 and 35 years participated in this study (age: 24 ± 4 years; height: 1.82 ± 0.07 m; mass: 85.7 ± 9.4 kg). Participants were required to be free from lower-limb injury in the 6 months prior to testing. Each participant provided informed consent prior to data collection. Ethical approval was obtained from Cardiff Metropolitan University ethics committee, with reference number PGR-3539.

### 2.2. Experimental Procedure

Participants completed a short warm-up consisting of slow running and stretching, and then performed the following three movements (in order): a bilateral drop jump from 30 cm, a unilateral drop jump from 20 cm, and a 90° pre-planned cut, following previously described protocols [21,25]. Briefly, during the drop jumps participants placed their hands on their hips and were told to roll from the step and upon hitting the ground, to jump as high as they could while spending as little time as possible on the force plate. For the bilateral drop jump, participants began with their feet approximately hip-width apart and landed with one foot on each of the force plates [21]. For the 90° pre-planned cut (hereafter referred to as the cut), participants were required to start at a distance of 5 m from the force plates, run as fast as possible toward the force plates, cut left or right while planting their contralateral foot on the force plate, and then accelerate away after changing direction [25]. Before the test trials were captured, participants underwent two sub-maximal practice trials of each movement. The right (dominant) leg for all participants was tested first, with a 30 s rest period between trials. Five valid attempts (determined by confirming a full-foot contact on the force plate) were recorded for each limb.

### 2.3. Biomechanical Data Collection

**Figure 1:**
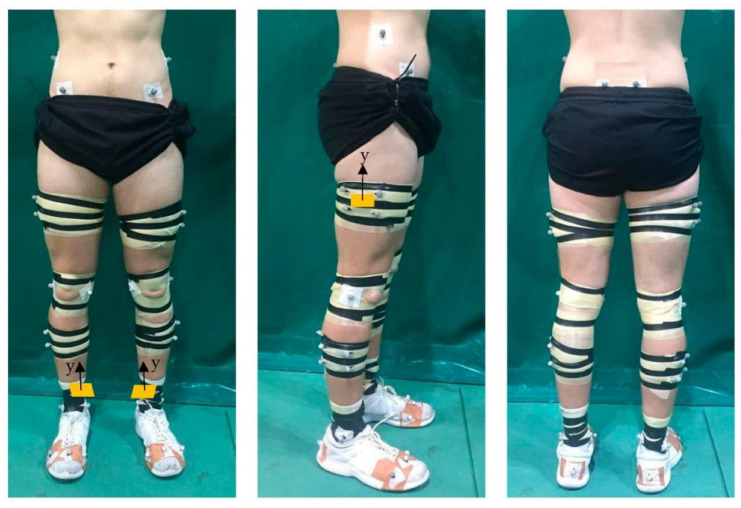
Example marker and IMU set up. Orange rectangles represent location of IMUs. IMU: inertial measurement unit.

All testing took place in the National Indoor Athletics Centre, Cardiff Metropolitan University. A 12-camera three-dimensional motion capture system (250 Hz; Vicon Motion Systems Ltd., Oxford, UK) was used to collect kinematic data. Two force platforms (1000 Hz; 9287CA, 90 × 60 cm, Kistler, Winterthur, Switzerland) were embedded in the ground to collect ground reaction forces (GRFs) and were synchronized to the Vicon system. During the trials, lower-body kinematic data were collected using a 38 reflective marker set attached to the participants’ skin bilaterally on the iliac crest, anterior and posterior superior iliac spine, lateral and medial femoral epicondyles, lateral and medial malleoli, first and fifth metatarsal heads, head of the second toe, and the calcaneus, in addition to technical clusters of four markers attached in the middle of the thigh and shank segments (Figure 1). Four IMUs (Blue Trident, Vicon Motion Systems Ltd., Oxford, UK) were attached bilaterally on the lateral mid-thigh on the rigid plate of the technical cluster and anteromedially over the distal tibia (Figure 1), with the y axes aligning with the longitudinal axes of the thigh and tibia, respectively. The position of the tibia marker was chosen to reduce soft tissue artefact [26,27]. Participants wore their own athletic footwear, and the reflective markers and IMUs were secured to the skin or to the shoe using tape. All markers and IMUs were applied by the same researcher to ensure accuracy and consistency throughout data collection.

Static and functional calibration trials were recorded of participants standing in the anatomical position and completing five body-weight squats and five leg swings on each side, respectively. Initial marker labelling and gap filling took place in Vicon Nexus (v.2.12.1, Oxford Metrics Inc., Oxford, UK). Data were exported to Visual3D (v.6, C-motion, Rockville, MD, USA), where static calibration trials were used to build a seven-segment (pelvis, thighs, shanks, and feet), 6 degrees-of-freedom kinematic model, and inverse kinematics were applied. Raw marker trajectories and GRF data used for inverse dynamic analysis calculations were filtered using a fourth-order low-pass Butterworth filter at 15 Hz [28]. Knee kinematics were expressed relative to the proximal segment (thigh) defined by an XYZ-ordered Cardan angle sequence [29]. The local segment coordinate systems and joint centres were established using an in-built Visual3D algorithm [30]. Segment inertial characteristics were estimated for each participant based on Dempster’s regression equations [31] and represented as geometric volumes [32]. Standard inverse dynamic analysis was used to calculate external knee moments. Knee moments were normalized to body mass [33]. All other biomechanical variables were calculated using these kinematic and kinetic data and exported to MATLAB (version R2022a, MathWorks Inc, Natick, MA, USA) and Excel (Microsoft Corporation, Redmond, WA, USA) for further processing and analysis.

### 2.4. Biomechanical Data Processing and Analysis

Sagittal plane knee kinematic and kinetic analyses were carried out for the braking phase of the bilateral and unilateral drop jumps and the cut. The braking phase was defined as the time between initial contact (determined as GRF > 20 N) to maximum knee flexion. Only data from the first landing (i.e., landing from the box) for the bilateral and unilateral drop jump were analyzed. Knee variables of interest were knee RoM, change in knee moment, and knee stiffness. Knee RoM was calculated as the magnitude of change from initial contact to maximum knee flexion, and change in knee moment was calculated between the same time points. Knee stiffness was determined as the ratio of change in knee moment to knee RoM.

### 2.5. IMU Data Processing and Analysis

The IMUs were synchronized with the Vicon system. The IMU’s raw capture rate was 225 Hz, which was then automatically up-sampled in Vicon Nexus to 1000 Hz using spherical linear interpolation. The variables of interest from the IMUs, angular velocity and acceleration, were measured using the gyroscope and accelerometer, respectively. Gyroscope data were filtered using a fourth-order low-pass Butterworth filter at 30 Hz, and accelerometer data were filtered using a fourth-order low-pass Butterworth filter at 60 Hz. Gyroscope and accelerometer data were then down-sampled to match the collection frequency of the motion capture system (250 Hz). The resultant angular velocities and accelerations in the braking phase (determined from the force and motion data) were analyzed. The resultants were calculated as the square root of the sum of each X, Y, and Z IMU axis squared. The peak resultant tibia and thigh angular velocities and accelerations were identified as the maximum value during the braking phase. The resultant area under the tibia and thigh angular velocity and acceleration curves were calculated by integrating the respective curves. The resultant angular velocity rate and acceleration rate were calculated by dividing the peak resultant value by the duration over which the peak occurred.

### 2.6. Statistical Analysis

The means and standard deviations (SDs) of either the first three trials or all five trials for each participant were computed. The right and left leg mean data were combined, resulting in 38 samples being included in the analysis. For statistical analysis, the Shapiro–Wilk test was used to test normality for all variables in each movement. Due to the non-normality of data, multiple Spearman’s correlations were run to determine the relationship between the gold-standard motion analysis and force-plate-derived knee variables (knee RoM, change in knee moment, and knee stiffness) and the IMU-derived metrics of peak angular velocity, area under the angular velocity curve, angular velocity rate, peak acceleration, area under the acceleration curve, and acceleration rate in each movement (bilateral and unilateral drop jump and the cut). Additionally, the strength of the correlations was compared between taking the mean of three trials and taking the mean of five trials. Correlations were reported as negligible (−0.3 > r_s_ < 0.3), weak (0.3 ≤ r_s_ < 0.5 or −0.3 ≥ r_s_ > −0.5), moderate (0.5 ≤ r_s_ < 0.7 or −0.5 ≥ r_s_ > −0.7), or strong (r_s_ ≥ 0.7 or r_s_ ≤ −0.7) [34]. Statistical analysis was performed using SPSS Statistics (SPSS 27, IBM). The level of significance was set at *p* ≤ 0.05.

## 3. Results

All correlations are presented in Table 1 and Table 2. Similar levels of correspondence were observed between taking the mean of three trials and taking the mean of five trials (Table 1 and Table 2). Therefore, the results discussed from now on are from taking the mean of three trials.

### 3.1. IMU-Derived Angular Velocities vs. Knee Variables

**Figure 2:**
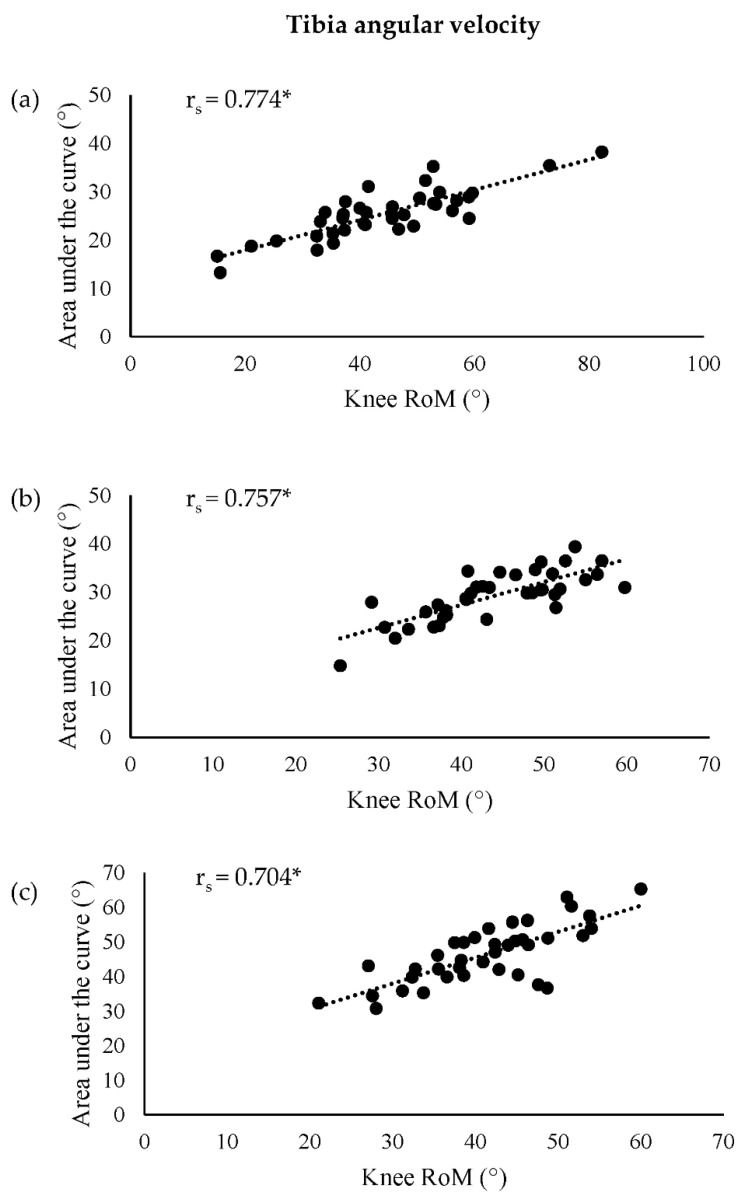
Correlations between knee RoM and area under the tibia angular velocity curve for the (**a**) bilateral drop jump, (**b**) unilateral drop jump, and (**c**) cut. * *p* ≤ 0.05. RoM: range of motion.

There was a significant strong positive relationship between knee RoM and the area under the tibia angular velocity curve during all movements (Figure 2). There were also significant moderate negative relationships between knee stiffness and the area under the tibia angular velocity curve in all movements (Table 1). For the unilateral drop jump, there was a significant moderate negative relationship between the change in knee moment and the area under the tibia angular velocity curve. In the bilateral drop jump, a significant moderate negative correlation was observed between knee stiffness and the area under the thigh angular velocity curve, as well as a significant moderate positive correlation between knee RoM and the area under the thigh angular velocity curve.

### 3.2. IMU-Derived Accelerations vs. Knee Variables

**Figure 3:**
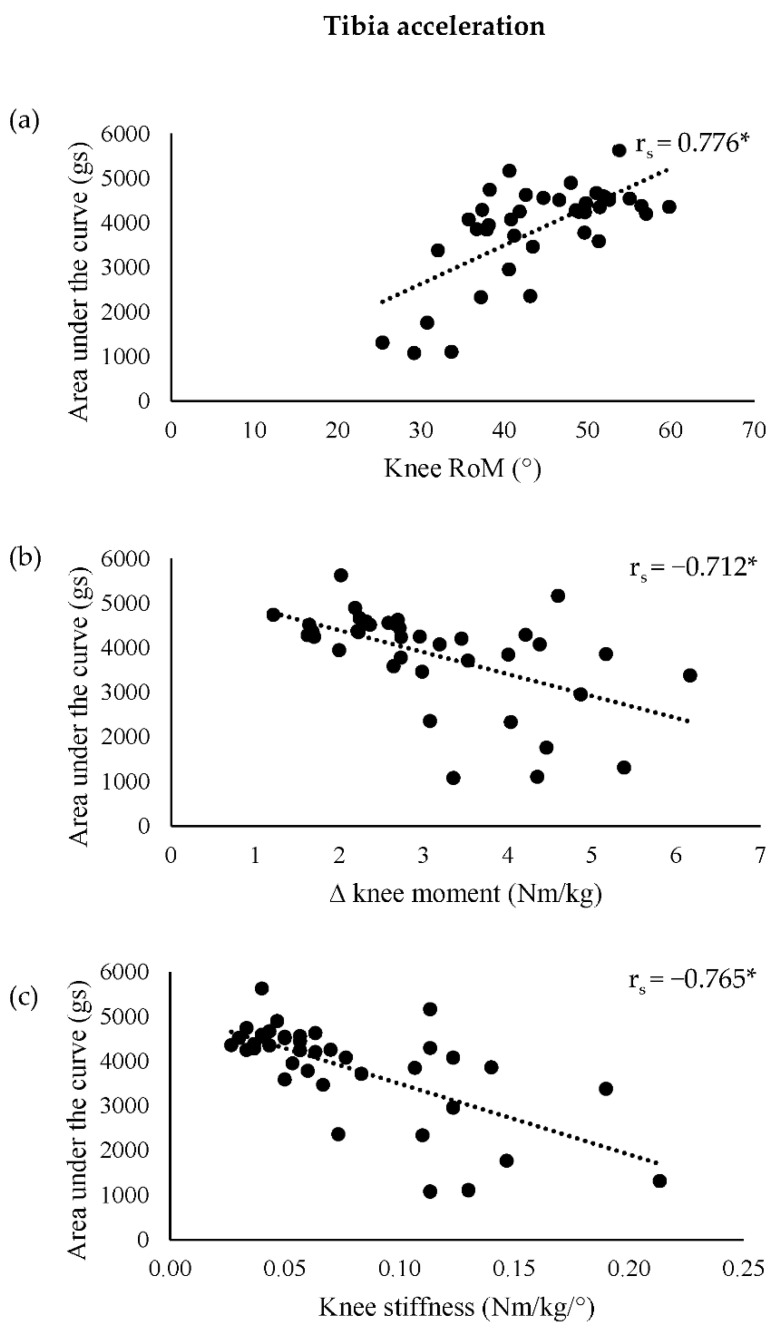
Correlations for the unilateral drop jump between (**a**) knee RoM, (**b**) ∆ knee moment, and (**c**) knee stiffness, and the area under the tibia acceleration curve. * *p* ≤ 0.05. ∆: change in; RoM: range of motion.

Significant strong correlations were observed in the unilateral drop jump between all knee variables and the area under the tibia acceleration curve (Figure 3). In addition, in the unilateral drop jump, there were significant moderate correlations between all knee variables and the tibia acceleration rate (Table 2). Significant moderate negative correlations were found in the bilateral drop jump between the change in knee moment and both the area under the tibia acceleration curve and peak tibia acceleration, as well as between knee stiffness and the area under the tibia acceleration curve. During the cut, significant moderate positive correlations were observed between both knee stiffness and change in knee moment, and the tibia acceleration rate.

## 4. Discussion

Given the high incidence rates of both primary and secondary ACL injuries in multidirectional field sports, there is a need to develop easily accessible methods that can be used as proxy measures to assess knee variables associated with ACL injury in the field. Inertial measurement unit-derived metrics may facilitate the ability of practitioners to monitor ACL injury risk more precisely in the field. Therefore, the aim of this study was to identify whether there were correlations between laboratory-derived knee variables (knee RoM, change in knee moment, and knee stiffness) and metrics derived from IMUs (angular velocities and accelerations) placed on the tibia and thigh, across a range of movements (bilateral and unilateral drop jumps and a cutting manoeuvre) performed in practitioner assessments to monitor ACL injury risk.

A strong positive relationship was observed between knee RoM and the area under the tibia angular velocity curve for all three movements. This indicates that greater angular displacement of the tibia is associated with larger knee RoM. When there is a smaller displacement of the tibia during braking, the tibia is displaced less anteriorly, i.e., in a more vertical position relative to the femur, and the knee is likely to be in a more extended position [35]. A more extended knee has been reported to increase anterior tibial shear force [36], anterior tibial translation [37,38,39,40], external knee extensor moments [41], knee abduction angle [42], external knee abduction moments [36,43], and tibial internal rotation [37,38,39,40,42], all of which have been suggested to strain the ACL. Consequently, increasing knee RoM is considered a safer movement strategy to prevent ACL injury. In support of this, larger knee RoM in healthy participants during landing from a drop jump was found to decrease peak anterior tibial shear [41], and would therefore be deemed beneficial to reduce the risk of ACL injury. As in this study a greater knee RoM was found to correlate with larger area under the tibia angular velocity curve values, it could be implied that greater area under the tibia angular velocity curve values in ACL injury monitoring assessments are indicative of reduced ACL injury risk. Therefore, the area under the tibia angular velocity curve could be used as a proxy measure for assessing knee RoM in bilateral and unilateral drop jumps, and a 90° cut, to facilitate the ability of practitioners to monitor ACL injury risk in the field.

Strong negative correlations were observed during the unilateral drop jump between both knee stiffness and change in knee moment, and the area under the tibia acceleration curve, whilst a positive correlation was found between knee RoM and the area under the tibia acceleration curve. Larger values of the area under the tibia acceleration curve are indicative of higher magnitudes of accelerations throughout the duration of the braking phase. This may be explained by Derrick’s [44] effective mass theory. Briefly, the effective mass theory proposes that the effective mass of the shank-foot complex is reduced by its uncoupling from the rest of the body through increased sagittal plane knee RoM. Subsequently, the lower effective mass can be accelerated more quickly throughout the braking phase, resulting in the larger area under the tibia acceleration curve values observed at increased knee RoM in this study. As a result of increased knee RoM and smaller changes in knee moment, a lower knee stiffness was reported and related to an increase in the magnitude of tibial acceleration during the braking phase in the unilateral drop jump. This differs from previous research, which found positive relationships between peak vertical tibial accelerations and knee stiffness, and a negative relationship with knee RoM [19]. However, Milner et al. [19] examined the initial loading phase during running, defined from initial contact to impact peak, and the relationship with peak axial tibia acceleration. Athletes utilize different movement and loading strategies when performing bilateral and unilateral drop jumps, and cutting manoeuvres compared with those used in running, which may explain the difference in the relationships observed between the two studies. The findings from this study suggest that the area under the tibia acceleration curve may be a useful proxy measure for practitioners to detect differences in knee stiffness, change in knee moment, and knee RoM in applied field-based settings, though only in a unilateral drop jump.

To confirm whether mean IMU metrics calculated from three trials were stable, the strength of correlations between taking the mean of three trials (existing ACL risk-monitoring protocols) against the mean of five trials were also assessed. Similar levels of correspondence with knee variables were reported between taking the mean of three trials and taking the mean of five trials (Table 1 and Table 2). This has implications for field-based assessments that seek to monitor ACL injury risk, as it demonstrates that a mean value based on three trials provides sufficient stability to evaluate relevant IMU-derived metrics as proxies for several knee variables.

As relationships were found between knee variables and IMU-derived metrics, this study sought to identify the location of the IMU (tibia or thigh) that demonstrated the strongest correlations, to help improve the efficiency of practitioner assessments. Subsequently, if practitioners only have the time and finances available to apply a single sensor to each lower limb, this study identified that, compared with those of the thigh-mounted IMU, the resultant angular velocities and accelerations derived from a tibial-mounted IMU are most strongly correlated with knee variables used to assess ACL injury risk. In addition, due to the number of athletes that require injury risk assessment, practitioners testing in the field are often constrained by time. As a result, combining all axes from a triaxial IMU to calculate resultant angular velocities and accelerations, as used in this study, would be beneficial since the orientation of the IMU does not have to be aligned to a specific axis, thus improving the repeatability of using an IMU in the field [45].

Some limitations must be addressed. Firstly, since the level of variation when performing movements likely displayed in ACL-reconstructed individuals could have made it difficult to detect any associations that may have existed, only healthy participants were assessed in this study. As a group of participants at risk of ACL injury or re-injury were not included for comparison, it is not possible to confirm whether IMU-derived proxy measures of knee variables could be used to identify individuals at higher risk of second ACL injury. Since correlations were observed between knee variables (knee RoM, change in knee moment, and knee stiffness) and angular velocities and accelerations derived from IMUs in healthy participants, future research could compare patients who have had an ACL reconstruction to non-injured controls to investigate the feasibility of using IMUs on ACL reconstructed individuals. This could ultimately lead to expediting the individual’s return to sport, as deficits observed in more realistic field-based settings (as opposed to the practitioner’s room/laboratory) could be better targeted during rehabilitation. Secondly, the position of the thigh-located IMUs coincided with the position of the rigid plates of the technical clusters. In an applied field-based setting, technical clusters would not be attached. Therefore, further work is needed to establish the validity of the measurements of thigh-located IMUs when they are placed on the skin. However, a thigh-located IMU is likely to have increased measurement artefacts due to wobbling masses. Finally, this study used motion and force data to identify the braking phase for the IMU-derived metrics. Using motion and force data to detect initial contact and maximum knee flexion to determine the beginning and end of the braking phase, respectively, would not be possible in the field. Accelerations from a shin-mounted accelerometer however have been used to identify initial contact during running [46]. It may also be possible to determine peak knee flexion from IMU-derived angular velocities, as peak knee flexion may coincide with an angular velocity of zero. Future research should focus on determining initial contact and maximum knee flexion events using IMU-derived metrics from a tibia-located IMU to define the braking phase, without the need for gold-standard equipment, in movements that practitioners would use to assess ACL injury risk.

## 5. Conclusions

The findings from this study suggest that it may be feasible to use IMU-derived angular velocities and accelerations as proxy measures of knee variables in movements included in practitioner assessments used to monitor ACL injury risk. Further research in this population would help to confirm whether these field-based IMU-derived metrics provide a suitable proxy measure for identifying individuals who display lower limb biomechanics that may be associated with an ACL injury. Specifically, the area under the tibia angular velocity curve may be used as a proxy measure for knee RoM in the bilateral and unilateral drop jumps, and the cut. The area under the tibia acceleration curve may be a useful proxy measure for practitioners wanting to detect differences in knee joint stiffness, change in knee moment, and knee RoM in an applied setting, but only in a unilateral drop jump. Finally, the resultant angular velocities and accelerations derived from a tibia-mounted IMU were most strongly correlated with knee variables associated with ACL injury, as opposed to those derived from a thigh-located IMU. Therefore, if practitioners were looking to apply only a single sensor on each lower limb, IMUs located on each tibia would be recommended.

## Figures and Tables

**Table 1:** Correlations between knee variables and IMU-derived angular velocities for all movements. Mean of 3 trials on the left side of the table. Mean of 5 trials on the right side of the table. Red cells denote weak correlations. Yellow cells denote moderate correlations. Green cells denote strong correlations.

			Knee Variables					
			Mean of 3 Trials			Mean of 5 Trials		
			Knee RoM (°)	∆ Knee Moment (Nm/kg)	Knee Stiffness (Nm/kg/°)	Knee RoM (°)	∆ Knee Moment (Nm/kg)	Knee Stiffness (Nm/kg/°)
Thigh	Max angular velocity (°/s)	Bilateral drop jump	0.065	−0.121	−0.131	0.159	−0.172	−0.207
		Unilateral drop jump	−0.129	0.152	0.16	−0.139	0.091	0.109
		Cut	0.340 *	0.380 *	−0.075	0.324 *	0.360 *	−0.094
	Area under the angular velocity curve (°)	Bilateral drop jump	0.682 *	−0.398 *	−0.641 *	0.761 *	−0.483 *	−0.705 *
		Unilateral drop jump	0.457 *	−0.352 *	−0.376 *	0.398 *	−0.312	−0.355 *
		Cut	0.418 *	0.173	−0.266	0.411 *	0.205	−0.258
	Angular velocity rate (°/s^2^)	Bilateral drop jump	−0.209	0.238	0.267	−0.047	0.198	0.181
		Unilateral drop jump	−0.167	0.235	0.213	−0.200	0.308	0.250
		Cut	0.184	0.289	0.022	0.109	0.363 *	0.120
Tibia	Max angular velocity (°/s)	Bilateral drop jump	0.096	−0.182	−0.177	0.007	−0.173	−0.152
		Unilateral drop jump	0.205	−0.158	−0.197	0.122	−0.166	−0.163
		Cut	0.292	0.380 *	−0.048	0.295	0.345 *	−0.055
	Area under the angular velocity curve (°)	Bilateral drop jump	0.774 *	−0.354 *	−0.671 *	0.766 *	−0.511 *	−0.747 *
		Unilateral drop jump	0.757 *	−0.552 *	−0.624 *	0.747 *	−0.547 *	−0.635 *
		Cut	0.704 *	−0.002	−0.558 *	0.700 *	0.078	−0.542 *
	Angular velocity rate (°/s^2^)	Bilateral drop jump	0.024	−0.137	−0.121	−0.114	0.016	0.008
		Unilateral drop jump	−0.080	0.052	0.054	−0.159	0.169	0.165
		Cut	−0.153	0.270	0.279	−0.076	0.278	0.270

* *p* ≤ 0.05. ∆: change in; RoM: range of motion.

**Table 2:** Correlations between knee variables and IMU-derived accelerations for all movements. Mean of 3 trials on the left side of the table. Mean of 5 trials on the right side of the table. Red cells denote weak correlations. Yellow cells denote moderate correlations. Green cells denote strong correlations.

			Knee Variables					
			Mean of 3 Trials			Mean of 5 Trials		
			Knee RoM (°)	∆ Knee Moment (Nm/kg)	Knee Stiffness (Nm/kg/°)	Knee RoM (°)	∆ Knee Moment (Nm/kg)	Knee Stiffness (Nm/kg/°)
Thigh	Peak acceleration (g)	Bilateral drop jump	−0.434 *	0.248	0.362 *	−0.416 *	0.301	0.361 *
		Unilateral drop jump	−0.347 *	0.248	0.321 *	−0.387 *	0.228	0.337 *
		Cut	0.186	0.318	0.001	0.152	0.316	−0.037
	Area under the acceleration curve (gs)	Bilateral drop jump	0.231	−0.326 *	−0.359 *	0.325 *	−0.344 *	−0.420 *
		Unilateral drop jump	0.076	−0.065	−0.039	0.029	−0.062	−0.038
		Cut	0.385 *	0.280	−0.136	0.362 *	0.225	−0.17
	Acceleration rate (g/s)	Bilateral drop jump	−0.371 *	0.343 *	0.369 *	−0.382 *	0.422 *	0.413 *
		Unilateral drop jump	−0.476 *	0.335 *	0.417 *	−0.457 *	0.320 *	0.392 *
		Cut	−0.187	0.421 *	0.331 *	−0.196	0.405 *	0.323 *
Tibia	Peak acceleration (g)	Bilateral drop jump	0.291	−0.521 *	−0.485 *	0.248	−0.465 *	−0.400 *
		Unilateral drop jump	0.134	−0.331 *	−0.294	0.020	−0.263	−0.222
		Cut	−0.378 *	0.264	0.380 *	−0.350 *	0.229	0.315 *
	Area under the acceleration curve (gs)	Bilateral drop jump	0.472 *	−0.524 *	−0.591 *	0.489 *	−0.556 *	−0.644 *
		Unilateral drop jump	0.776 *	−0.712 *	−0.765 *	0.688 *	−0.700 *	−0.724 *
		Cut	0.208	0.011	−0.181	0.200	−0.066	−0.287
	Acceleration rate (g/s)	Bilateral drop jump	0.276	−0.494 *	−0.459 *	0.241	−0.450 *	−0.427 *
		Unilateral drop jump	0.582 *	−0.618 *	−0.667 *	0.554 *	−0.588 *	−0.655 *
		Cut	−0.323 *	0.516 *	0.555 *	−0.338 *	0.538 *	0.591 *

* *p* ≤ 0.05. ∆: change in; RoM: range of motion.

## Data Availability

The data are available on reasonable request and due to restrictions, e.g., privacy or ethical.
